# Predictors of response to axicabtagene‐ciloleucel CAR T cells in aggressive B cell lymphomas: A real‐world study

**DOI:** 10.1111/jcmm.17550

**Published:** 2022-12-01

**Authors:** Lorenzo Iovino, Qian Vicky Wu, Jenna Voutsinas, Lorena Panaite, Erin Mullane, Ryan C. Lynch, Chaitra Ujjani, Stephen D. Smith, Ajay K. Gopal, Brian G. Till, Filippo Milano, Victor Chow, Jordan Gauthier, Cameron J. Turtle, David G. Maloney, Mazyar Shadman

**Affiliations:** ^1^ Clinical Research Division Fred Hutchinson Cancer Research Center Seattle Washington USA; ^2^ Department of Medicine University of Washington Seattle Washington USA

## Abstract

Chimeric antigen receptor T‐cell (CAR T) therapy has shown promising efficacy in relapsed and refractory diffuse large B cell lymphoma (DLBCL). While most patients undergo CAR T infusion with active disease, the impact of some clinical variables, such as responsiveness to the pre‐CAR T chemotherapy on the response to CAR T, is unknown. In this single‐institution study, we studied the impact of several pre‐CAR T variables on the post‐CAR outcomes. Sixty patients underwent apheresis for axicabtagene‐ciloleucel (axi‐cel) and 42 of them (70.0%) had primary refractory disease. Bridging therapy between apheresis and lymphodepletion was given in 34 patients (56.7%). After axi‐cel, the overall response rate was 63.3%. Responsiveness to the immediate pre‐CAR T therapy did not show a significant association with response to axi‐cel, progression‐free (PFS) or overall (OS) survival. Multivariable analysis determined that bulky disease before lymphodepletion was independently associated with inferior outcomes, and patients that presented with high‐burden disease unresponsive to immediate pre‐CAR T therapy had a dismal outcome. This data supports proceeding with treatment in CAR T candidates regardless of their response to immediate pre‐CAR T therapy. Interim therapeutic interventions should be considered in patients who have known risk factors for poor outcomes (bulky disease, high LDH).

## 1 INTRODUCTION

Immunotherapy using chimeric antigen receptor T cells (CAR T) has become a standard of care treatment for patients with diffuse large B‐cell lymphoma (DLBCL) and other aggressive histologies such as transformed follicular lymphoma (tFL), primary mediastinal lymphoma (PMBL) and high‐grade lymphomas (HGBL). There are currently three food and drug administration (FDA)‐approved products for patients with relapsed or refractory (R/R) disease after two lines of therapy. While most patients have active disease at the time of CAR T therapy, some may present with late relapses and low disease burden, whereas others present with progressive disease on chemotherapy that is given before CAR T. Bridging therapy (BT) is often utilized for the latter group as a debulking strategy to control disease until cell infusion.

## 2 PATIENTS AND METHODS

In this single centre, retrospective study, we included 60 consecutive adult patients presenting with relapsed/refractory DLBCL, tFL, PMBCL or HGBCL who were treated with axicabtagene ciloleucel (axi‐cel) after lymphodepletion with cyclophosphamide and fludarabine per standard guidelines^1^ at the University of Washington/Fred Hutchinson Cancer Research Center between 2018 and 2020. The study was approved by the Fred Hutchinson Cancer Research Center Institutional Review Board; all patients provided written, informed consent. The severity of cytokine release syndrome (CRS) and immune effector cell‐associated neurotoxicity syndrome (ICANS) was graded according to the American Society for Transplantation and Cellular Therapy (ASTCT) consensus criteria.^2^

Pre‐ and post‐CAR responses were defined by computed tomography (CT) or positron emission tomography (PET‐CT) scans according to the Lugano 2014 criteria.^3^ Pre‐CAR T responsiveness was defined as having achieved at least a partial response (PR) to the last cycle of therapy that patients received before LD. For patients who received BT, responsiveness was evaluated on the PET‐CT scan performed after the last cycle of BT compared with pre‐BT scans. Using the Kaplan–Meier method, overall survival (OS) was calculated from the date of CAR T‐cell infusion until the date of death from any cause or the date of last contact. Progression‐free survival (PFS) was measured from the date of CAR T‐cell infusion to the date of death from any cause, disease relapse or progression, or the date of last contact. A Cox proportional‐hazards model with a stepwise selection procedure was used to select prognostic covariates, based on their statistical significance (*p* < 0.05) on univariate analyses as well as clinical factors that have been associated with an impact on survival, such as BT and double hit/triple hit (DH/TH) status, and responsiveness to immediate pre‐CAR T therapy. Significant covariates from the univariate overall response (OR) model were included in the multivariable ORR analysis. All statistical analyses were performed using R (R Foundation for Statistical Computing).

## 3 RESULTS

### 3.1 Patient characteristics

#### 3.1.1 Baseline

Sixty pts (20 female) were included in this analysis. Patient characteristics are outlined in Tables 1 and 2. In first line, 48 patients received a R‐CHOP‐like regimen (80.0%), 9 patients received an R‐EPOCH‐like regimen (15.0%), and 3 patients (5.0%) received other types of therapy (one R‐Bendamustine, one rituximab alone, one R‐Magrath modified) (Table 1). Forty‐two patients (70.0%) were primary refractory (defined as no response to first‐line therapy or relapse in the first 12 months) and 16 patients (26.7%) had received a previous stem cell transplant (one received an allogeneic transplant). All patients received at least two lines of immune‐chemotherapy before lymphodepletion (LD). Twenty‐one patients (34.4%) received CAR‐T after failing two previous lines of therapy, 21 (34.4%) for R/R after three lines, and 19 (21.2%) after four or more lines of therapy. Overall, 11 patients (18.3%) had obtained at least a PR to the last line of therapy received before the referral to our immunotherapy service. Characteristics of the patients by the number of previous lines of therapy are outlined in Tables S1–S3.

**TABLE 1:** Baseline characteristics

	Overall (*N* = 60)
Age at the time of CAR T infusion	
Median (range)	62 (25.0–79.0)
Gender	
Female (%)	20 (33.3%)
Mutational status	
DH/TH (%)	16 (26.7%)
Only myc <40% (%)	44 (73.3%)
Primary diagnosis	
DLBCL (%)	44 (73.3%)
PMBCL (%)	2 (3.3%)
Transformed from indolent NHL (%)	14 (23.3%)
Number of treatment lines before CAR T, including ASCT	
Median (range)	3.0 (2.0–9.0)
Prior ASCT (%)	18 (30.0%)
ASCT to CAR T (months)	
Median (range)	22.0 (5.0–119.0)
First line of therapy	
R‐CHOP like (%)	48 (80.0%)
R‐EPOCH like (%)	9 (15.0%)
Other (%)	3 (5.0%)
Time from first line of therapy to CAR T (months)	
Median (range)	18.5 (5.0–267.0)
Response to first line of therapy	
Primary refractory (%)	42 (70.0%)
Relapsed (%)	18 (30.0%)
Months from response after first line of therapy and relapse	
Median (range)	57 (14.0–216.0)

Abbreviations: ASCT, autologous stem cell transplant; DH/TH, double hit/triple hit mutated; DLBCL: diffuse large B cell lymphoma; NHL, non‐Hodgkin lymphoma; PMBCL, primary mediastinal B cell lymphoma.

**TABLE 2:** Patient characteristics pre‐ and post‐CAR T infusion

Time from last therapy to leukoapheresis (weeks)	
Median (range)	8.5 (0.0–109.0)
Bridging therapy (%)	34 (56.7%)
Time from immediate pre‐CAR T therapy to lymphodepletion (weeks)	
Median (range)	4.1 (0.0–105.0)
Patients with available PET scan pre‐CAR T (%)	51 (85.0%)
ECOG Performance Status (%)	
0	21 (35.0%)
1	27 (45.0%)
2	9 (15.0%)
3	2 (3.0%)
4	1 (2.0%)
Response to immediate pre‐CAR T therapy (%)	
CR	3 (5.1%)
PR	11 (18.6%)
SD	21 (35.6%)
PD	24 (40.7%)
Pre‐CAR T LDH (UI/l)	
Median (range)	182.5 (103.0–1929.0)
Presence of bulky disease (%)	20 (33.3%)
Product of diameters of the bigger measurable lesion pre‐CAR T (cmxcm)	
Median (range)	13.97 (0.8–480.0)
Site of the largest measurable lesion pre‐CAR T	
Lymph nodes or spleen (%)	45 (75.0%)
Extranodal (%)	15 (25.0%)
CRS of any grade (%)	46 (76.7%)
Highest grade of CRS (%)	
1	16 (34.8%)
2	24 (52.2%)
3	4 (8.7%)
4	2 (4.3%)
ICANS (%)	31 (51.7%)
Highest grade of ICANS (%)	
1	4 (12.9%)
2	17 (54.8%)
3	8 (25.8%)
4	2 (6.5%)
Response at day + 28 post‐CAR T (%)	
CR	18 (32.1%)
PR	20 (35.7%)
SD	9 (16.1%)
PD	9 (16.1%)

Abbreviations: CR, complete response; CRS: cytokine release syndrome; ECOG, Eastern Cooperative Oncology Group; ICANS, Immune effector cell‐associated neurotoxicity syndrome; LDH, lactate dehydrogenase; PD, progression disease; PR, partial response; SD, stable disease.

#### 3.1.2 Pre‐CAR T

The median time from last therapy to leukapheresis (LA) was 8.5 weeks (range 0–109) and the median time from LA to LD was 23 days (range 14–45) (Table 2). The median Eastern Cooperative Oncology Group (ECOG) performance status was 1. Before LD, 20 patients (33.3%) presented with bulky disease, defined as presence of a lesion with at least one of the diameters exceeding 7 cm^4^; the product of the two longest diameters of the largest measurable lesion had a median value of 13.97 cm^2^ (0.8–480.0 cm^2^). Median lactate dehydrogenase (LDH) was 182.5 UI/L (103.0–1929.0 UI/L), with 25 patients (41.7%) having elevated LDH (>210 UI/L). In 15 patients (25.0%), the site of the largest lesion was extranodal. Thirty‐four patients (55.7%) received a BT between LA and LD, with a median of one cycle (range 1–2); the drugs used for BT are outlined in Table S2. Fifteen patients (25.0%) had received some type of targeted immunotherapy before undergoing LA for the manufacturing of axi‐cel: twelve out of them (80.0%) received immunotherapy targeting molecules other than CD19. Four among these 15 patients (26.7%) had received a previous different type of CAR T product: three an anti‐CD19 CAR and one an anti‐CD20 CAR T product. More details about previous targeted immunotherapies are included in Table S3. Overall, between previous lines of therapy and BT, 13 patients (21.7%) received polatuzumab vedotin before CAR T infusion. The median time from the last therapy, including BT, to lymphodepletion was 4.1 weeks (range 0–105). No patients (14.8%) responded to BT. Overall, response to the immediate pre‐CAR T therapy, including BT, was evaluable in 59 patients. In details: complete response (CR) was achieved in three patients (5.1%); partial response (PR) in 11 patients (18.6%); stable disease (SD) in 21 patients (35.6%); and progressive disease (PD) in 24 patients (40.7%). Fifty‐eight patients (96.7%) received a full dose of LD.

The 21 patients who received axi‐cel after two lines of therapy represented a more homogeneous population: 16 of them were primary refractory (76.2%); one (4.8%) had a history of transformed lymphoma, and six (28.6%) had failed a second line containing ASCT. Five patients (23.8%) had responded to second‐line therapy; median time from second‐line therapy to LA was 20 weeks for the responders (range 3–27) and 3.5 weeks (range 2–21) for non‐responders. Nine patients (42.9%) received BT between LA and LD: two out of the five responders (40.0%) and seven out of the 16 non‐responders (43.8%). Two out of two responders to second‐line therapy who had BT showed a response to BT (100%), whereas two out of seven non‐responders (26.9%) responded to BT. More data are shown in Tables S1–S4.

#### 3.1.3 Post‐CAR T

CRS occurred in 46 patients (76.7%), with CRS grade ≥3 occurring in 6 patients (10.0%). Thirty‐one patients (51.7%) developed ICANS, with grade ≥3 in 10 patients (32.3%). Twenty‐nine patients (48.3%) developed both CRS and ICANS (Table 2).

Fifty‐six patients were evaluable at the day +28 PET‐CT scan: eighteen patients (32.1%) obtained a CR, and 20 (35.7%) had a PR at day +28, with an overall response rate (ORR) of 67.8% (66.3% on the total of the patients enrolled in this study). Of the 38 patients who had obtained a CR or a PR at day +28, eight (21%) subsequently developed relapse or progression of disease, with a median time of relapse of 8.45 months (range 1.70–16.20). Among the 20 patients with SD at day +28, 6 (33%) converted to CR; none of the nine patients in PR at day +28 showed progression of disease during the follow‐up, five of them (33%) having converted to a CR at the last follow‐up. Finally, none of the 9 patients in PD at day +28 obtained any subsequent response.

At the time of the most recent follow‐up visit, 40 patients (66.7%) were alive; among the other 20 patients, three (15.0%) died due to therapy‐related toxicity, and 17 (85.0%) due to progression of disease. With a median follow‐up time of 4.78 months (range 0.96–31.1), the estimated median OS was 13.7 months and the estimated median PFS was 8.8 months.

#### 3.1.4 Impact of responsiveness to immediate pre‐CAR therapy on outcomes

**FIGURE 1:**
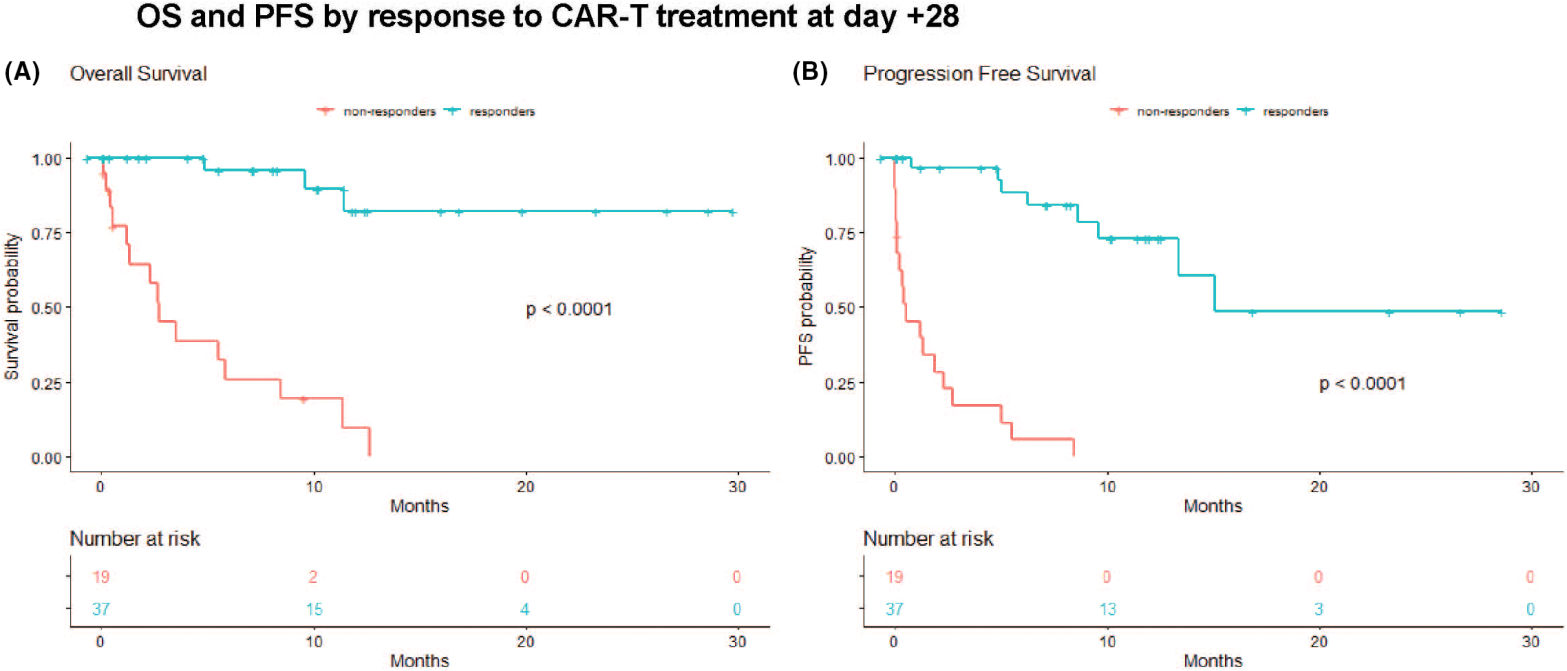
Kaplan–Meier curves comparing outcomes by response to CAR T at day +28. Responders: complete remission or partial remission, Non‐responders: stable disease or progression disease. (A) PFS. (B) OS. Only the 56 patients with an available re‐staging at day +28 were included in this analysis.

**FIGURE 2:**
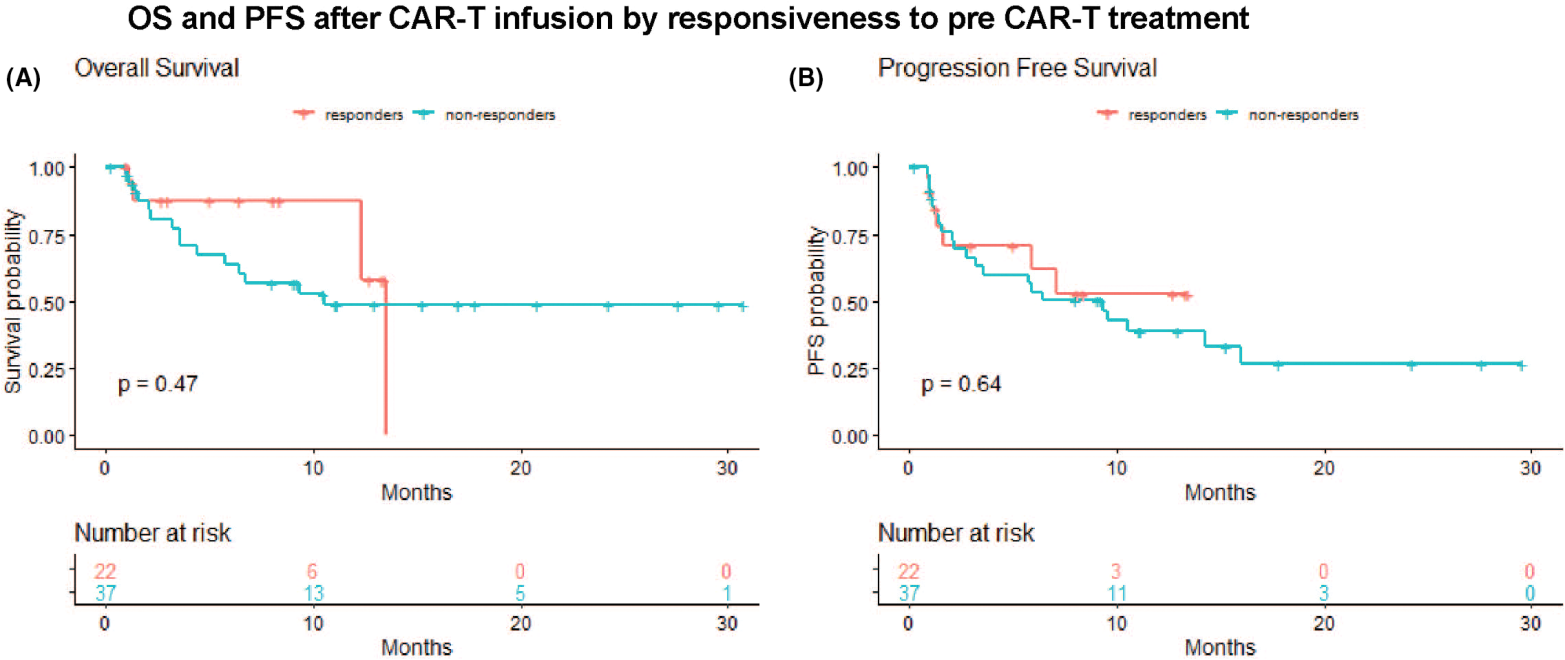
Kaplan–Meier curves comparing outcomes by response to immediate pre‐CAR T therapy. Responders: complete remission or partial remission, Non‐responders: stable disease or progression disease. (A) PFS. (B) OS. Only the 59 patients with an available re‐evaluation after the immediate pre‐CAR T therapy were included in this analysis.

We performed univariate analysis to identify baseline and therapy‐related factors that were associated with OS and PFS and included them in the subsequent multivariable analyses. Variables considered and results are outlined in Tables S4–S7. Univariate analyses demonstrated inferior OS in patients with pre‐LD low haematocrit (*p* = 0.013), low platelets (*p* = 0.002), high LDH (*p* = 0.018), presence of bulky disease (*p* = 0.016) and higher product of diameters of the largest measurable lesion (*p* = 0.025). Failure to achieve a CR or PR on day +28 was associated with inferior OS (*p* < 0.001) (Figure 1a) and PFS (*p* < 0.001) (Figure 1b). In univariate analysis, we did not observe any association between response to the last therapy before CAR T, this including both pre‐arrival therapy and BT, and OS (Figure 2a), PFS (Figure 2b), or response at day +28.

The multivariable analysis also confirmed that responsiveness to the immediate chemo‐immunotherapy administered pre‐CAR T was not associated with any of the clinical outcomes (Table 3). Variables that were independently associated with worse OS were as follows: elevated LDH (hazard ratio [HR]: 10.45; 95% confidence interval [CI], 1.99–55.02; *p* = 0.001), low haematocrit (HR: 0.78; 95% CI: 0.68–0.90; *p* = 0.001), having received BT (HR: 0.29; 95% CI: 0.09–0.91; *p* = 0.033), and the DH/TH status (HR: 9.08; 95% CI: 2.15–38.31; *p* = 0.003). Lower pre‐CAR haematocrit (HR: 0.91; 95% CI: 0.84–1.00; *p* = 0.046) and higher LDH (HR: 5.14; 95% CI: 1.11–23.88; *p* = 0.037) were associated with worse PFS. None of these variables were significantly associated with higher chance of obtaining a response at day +28. The product of diameters of the biggest lesion was not associated with OS or PFS after adjusting for other variables but remained significantly associated with day +28 response (Table S8).

**TABLE 3:** Association of pre‐CAR T treatment with post‐CAR‐CAR T OS, PFS, and response to CAR T in a multivariable analysis

Prognostic factors	OS		PFS		ORR	
	HR (95% CI)	*p*	HR (95% CI)	*p*	HR (95% CI)	*p*
Response to immediate pre‐CAR T therapy	1.79 (0.57–5.58)	0.315	2.27 (0.80–6.42)	0.124	0.90 (0.18–5.15)	0.898
Low HCT pre‐CAR T	0.78 (0.68–0.90)	**0.001**	0.91 (0.84–1.00)	**0.046**	1.15 (1.00–1.35)	0.060
Elevated LDH pre‐CAR T	10.45 (1.99–55.02)	**0.006**	5.14 (1.11–23.88)	**0.037**	0.12 (0.00–1.67)	0.140
Bridging therapy	0.29 (0.09–0.91)	**0.033**	0.61 (0.25–1.47)	0.269	1.94 (0.49–8.47)	0.355
DH/TH	9.08 (2.15–38.31)	**0.003**	1.09 (0.42–2.86)	0.860	2.07 (0.55–7.97)	0.279

*Note*: *p* values in bold are statistically significant.

Abbreviations: DH/TH, double hit/triple hit mutated; HCT, haematocrit; HR, hazard ratio; LDH, lactate dehydrogenase; ORR, overall response rate; OS, overall survival; PFS, progression‐free survival.

**FIGURE 3:**
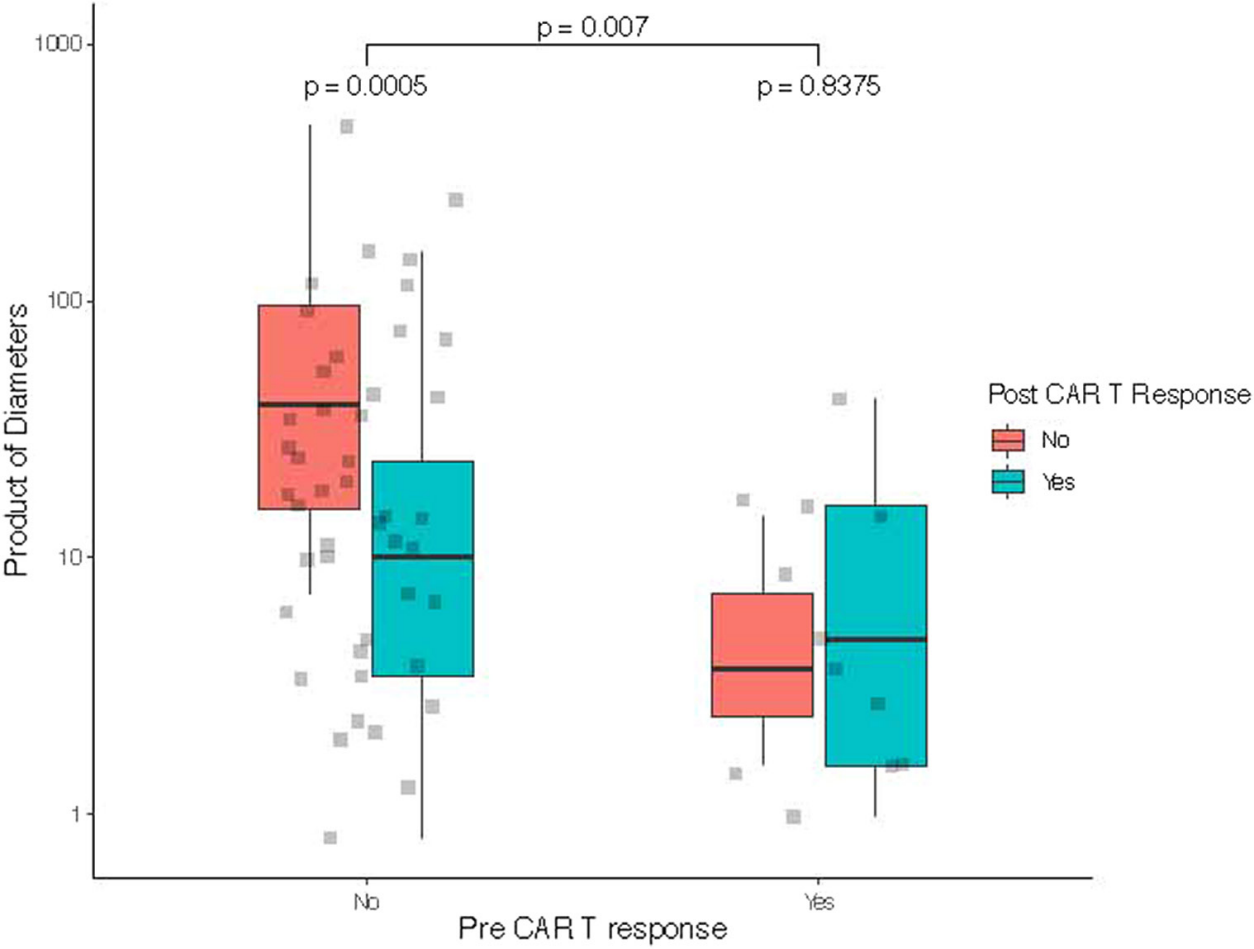
Product of diameters of the largest measurable lesion (expressed on the y axis as cm^2^) in patients who achieved at least a partial response to immediate pre‐CAR T therapy (box plots on the right of the *x* axis, “yes”) and patients who did not (box plots on the left of the *x* axis, “no”). Patients were then divided according to the response after CAR T therapy (Responders: blue box; non‐responders: red box). Brackets above the box‐and‐whisker plots indicate comparators in two‐tailed *t*‐test for independent samples.

Non‐responders to immediate pre‐CAR T therapy showed a higher tumour burden as expressed by product of diameters when compared to the responders (*p* = 0.0007). Response to CAR T was significantly associated (*p* = 0.0005) with a lower product of diameters pre‐LD among non‐responders to immediate pre‐CAR T therapy (Figure 3).

## 4 DISCUSSION

Early response to CAR T therapy is associated with better outcomes and prolonged survival, but little is known about other pre‐existing factors that are associated with negative outcomes. Responsiveness to chemotherapy is a critical factor when assessing patients with relapsed and refractory DLBCL for autologous stem cell transplant (ASCT). Patients with chemotherapy‐refractory disease are considered ineligible for ASCT because of the poor historical outcomes.^5^, ^6^, ^7^, ^8^, ^9^ One of the questions of this real‐world study was to investigate whether unresponsiveness to chemotherapy prior to CAR T infusion is a potential risk factor for poor response after CAR T. Our findings suggest that responsiveness to prior chemotherapy is not associated with post‐CAR T outcomes. While these results are not surprising from a biological standpoint and can be explained by the mechanism of action of CAR T therapy when compared to traditional chemotherapy and transplant, there are clinical implications from these findings. Our data supports timely utilization of CAR T therapy in eligible patients even with immediate chemo‐refractory disease without a need to use alternative therapies solely for disease stabilization purposes. In our series, many patients had chemo‐refractory disease at the time of leukapheresis; more than one‐third of patients received axi‐cel after failing a second‐line of therapy, this group representing a more homogeneous population to look at to address the question of this study. Even if our sample size is too small to perform a meaningful statistical analysis on this group, we could notice that patients who relapsed after having responded to second‐line therapy tended to respond also to BT. However, as outlined in Table S4, patients of this group who were chemo‐refractory to immediate pre‐CAR T therapy still responded to axi‐cel at day +28 in more than 80% of the cases. Larger studies specifically designed on a more homogeneous population and including other types of commercial CAR T products could help in giving a clear answer.

Our results confirm that bulky disease is an independent risk factor and that non‐responsive patients that present with high‐burden disease have a dismal outcome. We also observed an association between lack of response to pre‐CAR T chemo and post‐CAR T outcomes in patients with bulky disease. For defining bulky disease, we chose the cut‐off of 7 cm: the definition of bulky disease remains arbitrary and varies between 5 and 10 cm, depending on the studies and the cooperative groups.^10^, ^11^ However, in our analysis, we also considered the product of the diameters of the largest measurable lesion pre‐CAR T as a continuous variable, and that also showed to have a significant impact on the prognosis, especially in the chemo‐refractory setting. Having high disease burden is a predictor of poor outcomes in CAR T therapy^12^, ^13^ that may be a consequence of peculiar tumour vascularization^14^ and suboptimal infiltration and activation of the CAR T cells. Our data cannot suggest a clear cut‐off value based on the product of diameters of the largest measurable lesion, but other measures of tumour burden, such as total metabolic tumour volume (TMTV) estimated by PET‐CT.

Elevated pre‐LD LDH values have been described as negative prognostic factors for DLBCL patients receiving axi‐cel^13^, ^15^: our data confirm this association. LDH and TMTV are both parameters related to disease activity and lymphoma burden and have been demonstrated to predict early relapse after CAR T, either when they are elevated at the time of decision to proceed to CAR T‐cell therapy, or just before starting LD.^13^

Haematological toxicity is a common finding in cancer patients. Anaemia, either defined as haemoglobin or haematocrit values below the normal range, is a common side effect of axi‐cel and LD.^16^ To the best of our knowledge, a negative impact of pre‐LD haematocrit levels on post‐CAR T outcomes has not been described yet. In our series, low haematocrit did not correlate directly with other potential causes of anaemia, namely: creatinine clearance; lymphoma infiltration of the bone marrow; number of previous lines of therapies; previous ASCT; and time from the last pre‐LD therapy (data not shown). However, a study showed that elevated ferritin levels, which are usually associated with inflammatory anaemia, were associated with worse post‐CAR T outcomes.^13^ Another recent real‐world study showed that poor expansion of axi‐cel was significantly correlated with low platelet counts at the time of LD,^17^ a parameter that we found being associated with lower OS only in univariate analysis.

The incidence of non‐haematological toxicities of axi‐cel (CRS and ICANS) was comparable to other real‐world experiences with axi‐cel.^18^, ^19^ We did not find a statistical association between BT and higher incidence of ICANS, even if we did observe a higher incidence of ICANS among the patients who received BT (55.9%) compared with the patients who did not (46.1%, data not shown), as other authors have previously described.^20^

If these findings are confirmed in other series, they support the use of CAR T therapy in patients with chemo‐refractory disease, whereas other risk factors may instead be considered. For patients with bulky disease, it may be reasonable to reduce the disease bulk before CAR T therapy. With the recent introduction of novel approved or investigational therapeutic agents for lymphoma, including monoclonal antibodies,^21^ antibody‐drugs conjugates (ADCs),^22^, ^23^, ^24^, ^25^ bispecific antibodies^26^, ^27^, ^28^ and other targeted therapy agents,^29^, ^30^, ^31^, ^32^ such a strategy may be more feasible in the current era. Our data also suggest that there is a group of patients who are at very high risk of not achieving a CR post‐CAR T. Patients with concurrent bulky disease, elevated LDH levels, low haematocrit, and with chemo‐refractory disease should probably not be offered axi‐cel, preferring instead investigational drugs and cellular immunotherapies.

Our study is limited given the sample size and its retrospective nature and the fact that the sample size and heterogeneity may have limited our ability to fully assess the association chemosensitivity and response. In addition, radiographic assessments were not performed at standardized timepoints. Longer follow‐up is necessary to better understand post‐CAR T outcomes. However, given the known association between the response at day +28 and long‐term remissions,^16^, ^33^ the lack of impact of chemo‐responsiveness on the OS and PFS post axi‐cel is informative and at minimum hypothesis generating.

In conclusion, this study highlights the importance of proceeding with CAR T infusion, if possible, even in patients who are chemo‐refractory. Bulky disease is negative prognostic factor, especially in chemo‐refractory patients, and interim therapeutic approaches are suggested in those patients.

## AUTHOR CONTRIBUTIONS

**Lorenzo Iovino:** Conceptualization (lead); data curation (lead); formal analysis (equal); methodology (equal); writing – original draft (lead). **Qian Vicky Wu:** Data curation (lead); formal analysis (lead); methodology (lead); validation (equal). **Jenna M. Voutsinas:** Data curation (equal); formal analysis (lead); methodology (lead); software (equal); validation (equal); visualization (equal). **Lorena Panaite:** Visualization (equal); writing – review and editing (equal). **Erin Mullane:** Validation (equal); visualization (equal); writing – review and editing (equal). **Ryan Christopher Lynch:** Validation (equal); visualization (equal); writing – review and editing (equal). **Chaitra Ujjani:** Validation (equal); writing – original draft (equal); writing – review and editing (equal). **Stephen D Smith:** Validation (equal); visualization (equal); writing – review and editing (equal). **Ajay K. Gopal:** Supervision (equal); validation (equal); visualization (equal); writing – review and editing (equal). **Brian G. Till:** Validation (equal); visualization (equal); writing – review and editing (equal). **Filippo Milano:** Validation (equal); visualization (equal); writing – review and editing (equal). **Victor Chow:** Visualization (equal); writing – review and editing (equal). **Jordan Gauthier:** Visualization (equal); writing – review and editing (equal). **Cameron J. Turtle:** Visualization (equal); writing – review and editing (equal). **David G. Maloney:** Validation (equal); visualization (equal); writing – review and editing (equal). **Mazyar Shadman:** Conceptualization (lead); data curation (lead); funding acquisition (lead); resources (lead); supervision (lead); writing – original draft (equal); writing – review and editing (lead).

## CONFLICT OF INTEREST

LI, VW, JV, LP, EM, RCL, FM, VC and JC have no relevant conflicts of interest to declare. CSU: Consulting: atara, abbvie, Pharmacyclics, beigene, Epizyme, incyte, lilly, astrazeneca, Janssen, morphosys, ADC therapeutics, TG therapeutics. Research – abbvie, Pharmacyclics, lilly, astrazeneca, adaptive biotechnologies. BT: patent/royalties, advisory board, and research funding from Mustang Bio. DGM reports research funding from Bristol‐Myers Squib and Kite, a Gilead Company; rights to royalties from Fred Hutch for patents licensed to Bristol‐Myers Squibb; honoraria for advisory boards from Amgen, Bristol‐Myers Squibb, Genentech, Gilead Sciences, Incyte, Janssen, Kite, a Gilead Company, Legend Biotech, MorphoSys, Mustang Bio, Novartis, Pharmacyclics, and Umoja; has participated on a data safety monitoring board or steering committee for Bioline RX, Bristol‐Myers Squibb, and Genentech; has held a leadership role at Bristol‐Myers Squibb and Genentech; and has had honoraria and stock options from A2 Biotherapeutics and Navan Technologies, Inc. CJT received research funding from Juno Therapeutics, Nektar Therapeutics, AstraZeneca, TCR2 Therapeutics; is a member of scientific advisory boards for Precision Biosciences, Eureka Therapeutics, Caribou Biosciences, T‐CURX, Myeloid Therapeutics, ArsenalBio, and Century Therapeutics; has served on ad hoc advisory boards for Nektar Therapeutics, Allogene, Asher Biotherapeutics, PACT Pharma, Astra Zeneca; has stock options for Precision Biosciences, Eureka Therapeutics, Caribou Biosciences, Myeloid Therapeutics, ArsenalBio; and has patents licensed or optioned to Juno Therapeutics. MS: consulting, Advisory Boards, steering committees, and data safety monitoring committees: Abbvie, Genentech, AstraZeneca, Sound Biologics, Pharmacyclics, Verastem, ADC Therapeutics, Beigene, Cellectar, Bristol‐Myers Squibb, Morphosys, TG Therapeutics, Innate Pharma, Kite Pharma, Adaptive Biotechnologies, Epizyme, and Atara Biotherapeutics; Research Funding: Mustang Bio, Celgene, Bristol‐Myers Squibb, Pharmacyclics, Gilead, Genentech, Abbvie, TG Therapeutics, Beigene, AstraZeneca, and Sunesis.

## Supporting information


Table S1–S8

## Data Availability

The data that supports the findings of this study are available in the supplementary material of this article
