# Supplementary material for: Predictors of response to axicabtagene‐ciloleucel CAR T cells in aggressive B cell lymphomas: A real‐world study
Source: J Cell Mol Med. 2022 Dec 1;26(24):5976–83. doi: 10.1111/jcmm.17550 (PMC9753434; doi:10.1111/jcmm.17550)
Supplement: Supplementary file 1 — Table S1–S8 [file JCMM-26-5976-s001.docx]

**Supplemental Table 1. Patients characteristics at the time of leukapheresis (LA)**

| **Number of prior lines of therapy** | **Number of patients (%)** | **Response to the last line of therapy pre LA (%)** | **History of transformed lymphoma** | **Patients with bulky disease (%)** | **Prior ASCT (%)** | **Patients who received BT (%)** | **Response to BT (%)** |
| --- | --- | --- | --- | --- | --- | --- | --- |
| **2** | 21 (34.4%) | 5 (23.8%) | 1 (4.8%) | 5 (23.8%) | 6 (28.6%) | 9 (42.9%) | 4 (44.4%) |
| **3** | 21 (34.4%) | 4 (19.0%) | 3 (14.3%) | 9 (42.9%) | 7 (33.3%) | 14 (66.7%) | 2 (14.3%) |
| **4** | 6 (10.0%) | 0 (0.0%) | 1 (16.7%) | 1 (16.7%) | 1 (16.7%) | 4 (66.7%) | 2 (50.0%) |
| **>4** | 13 (21.2%) | 2 (15.3%) | 8 (61.5%) | 6 (46.2%) | 4 (30.8%) | 7 (53.8%) | 1 (7.7%) |
| **Overall** | **61 (100.0%)** | **11 (18.0%)** | **14 (23.3%)** | **20 (31.1%)** | **18 (30.0%)** | **34 (55.7%)** | **9 (14.8%)** |

Supplemental table 1. LA: leukapheresis. ASCT: autologous stem cell transplant. BT: bridging therapy. “Response to the last line of therapy pre LA” was evaluated on the PET-CT or CT scan performed after the last therapy received prior to the arrival at the immunotherapy service. “Response to BT” was evaluated only in patients who received BT by comparing the last pre-BT scan to the scan performed after the last cycle of BT. Patients were considered responders if obtained at least a partial response.

**Supplemental Table 2: type of bridging therapy (BT)**

| **Type of BT (%)** |  |
| --- | --- |
| **CHOP-like** | 3 (8.8%) |
| **Bendamustine-containing** | 4 (11.7%) |
| **Gemcytabine-containing** | 8 (23.5%) |
| **Polatuzumab vedotin-containing** | 11 (32.3%) |
| **Ibrutinib** | 2 (5.9%) |
| **Venetoclax** | 1 (2.9%) |
| **RICE** | 2 (5.9%) |
| **PBR** | 6 (17.6%) |
| **Rituximab-containing** | 24 (70.6%) |

Supplemental table 2. Type of bridging therapy (BT) received by 34 patients of our cohort.

**Supplemental Table 3: type of previous targeted immunotherapy**

| **Type of immunotherapy (%)** |  | **Type of product** |
| --- | --- | --- |
| **Previous CAR product** | 4 (26.7%) | 1 EGFRT+ CAR (anti-CD19)  1 tisagenlecleucel (anti-CD19)  1 JCAR014 (anti-CD19)  1 anti-CD20 CAR |
| **Polatuzumab vedotin** | 4 (26.7%) |  |
| **TTI-521 (anti-CD47)** | 1 (6.7%) |  |
| **Anti-4-IBB antibody** | 1 (6.7%) |  |
| **Anti-CD20 antibodies** | 3 (20.0%) | 1 Ofatumumab  1 Tositumomab  1 Ublituxomab |
| **Anti-CD3/anti-CD20 bispecific antibody** | 1 (6.7%) |  |
| **Anti-CD22 antibody** | 1 (6.7%) |  |

Supplemental table 3. Type of targeted immunotherapy (excluding rituximab) received by 15 patients of our cohort previous to the leukapheresis.

**Supplemental Table 4. Characteristics of the 21 patients who received axi-cel as a third line**

| \| Primary Refractory (%) \| 16 (76.2%) \|  \| \| --- \| --- \| --- \| \| 2nd Line Regimen \| **Patients (%)** \| **Response (%)** \| \| *RICE* \| 11 (52.4%) \| 3 (27.3%) \| \| *R-GCD* \| 4 (19.0%) \| 1 (25.0%) \| \| *R-GDP* \| 3 (14.3%) \| 1 (33.3%) \| \| *Other* \| 3 (14.3%) \| 0 (0%) \| \| *Total* \| 21 (100.0%) \| 5 (23.8%) \| \| Weeks from 2nd line to LA (range) \| *Responders to 2nd line* \| 20 (3-27) \| \|  \| *Non-responders to 2nd line* \| 3.5 (2-21) \| \| Bridging Therapy (BT) (%) \| 9 (42.9%) \| **Response (%)** \| \| *Responders to 2nd line* \| 2 (40.0%) \| 2 (100.0%) \| \| *Non-responders to 2nd line* \| 7 (43.8%) \| 2 (26.9%) \| \| Response To CAR T at Day +28 \| *Responders to 2nd line* \| 4/4 (100%) \| \| (%) \| *Non-responders to 2nd line* \| 13/16 (81.3%) \| \|  \| *Responders to the immediate pre-CAR therapy* \| 6/6 (100%) \| \|  \| *Non-responders to the immediate pre-CAR therapy* \| 11/14 (81.3%) \| |
| --- | --- | --- | --- | --- | --- | --- | --- | --- | --- | --- | --- | --- | --- | --- | --- | --- | --- | --- | --- | --- | --- | --- | --- | --- | --- | --- | --- | --- | --- | --- | --- | --- | --- | --- | --- | --- | --- | --- | --- | --- | --- | --- | --- | --- | --- | --- | --- | --- |

Supplemental table 2. LA: leukapheresis. BT: bridging therapy. Response to the 2nd line of therapy was evaluated on the PET-CT or CT scan performed for assessing the response after the 2^nd^ line of therapy. “Response to BT” was evaluated only in patients who received BT by comparing the last pre-BT scan to the scan performed after the last cycle of BT. Response to CAR-T was assessed at the day +28 PET-CT scan. Patients were considered responders if obtained at least a partial response.

**Supplemental Table 5. Univariate analysis for factors impacting OS**

***Pre-treatment***

| **Variable** | **HR** | **P value** | **HR (95% CI)** | |
| --- | --- | --- | --- | --- |
| Age | 0.98 | 0.344 | 0.95 | 1.02 |
| Sex | 0.72 | 0.482 | 0.29 | 1.81 |
| DH/TH | 2.18 | 0.148 | 0.76 | 6.29 |
| Diagnosis | 1.46 | 0.450 | 0.55 | 3.86 |
| Previous lines of treatment | 1.09 | 0.455 | 0.88 | 1.35 |
| Previous ASCT | 0.98 | 0.963 | 0.35 | 2.70 |
| Months to ASCT to CAR T | 0.99 | 0.612 | 0.96 | 1.02 |
| First line: R-CHOP vs R-EPOCH | 0.67 | 0.525 | 0.19 | 2.33 |
| First line: R-CHOP vs other | 1.90 | 0.540 | 0.24 | 14.89 |
| Months from first line to CAR T | 0.99 | 0.180 | 0.98 | 1.00 |
| Primary refractory disease | 0.44 | 0.188 | 0.13 | 1.50 |
| Time to relapse after first line | 1.01 | 0.326 | 0.99 | 1.04 |
| Weeks from last therapy to LA | 0.99 | 0.511 | 0.97 | 1.02 |
| Weeks from last therapy to LD | 0.80 | 0.462 | 0.45 | 1.44 |
| Bridging therapy | 0.74 | 0.517 | 0.30 | 1.83 |
| Novel drugs before CAR T | 0.87 | 0.765 | 0.34 | 2.19 |
| Previous use of polatuzumab vedotin | 2.25 | 0.354 | 0.41 | 12.47 |
| Previous infusion of a CAR T product | 0.44 | 0.197 | 0.13 | 1.52 |
| Number of bridging cycles | 1.11 | 0.793 | 0.52 | 2.38 |
| WBC pre-CAR T | 0.50 | 0.285 | 0.14 | 1.79 |
| ANC pre-CAR T | 0.66 | 0.454 | 0.22 | 1.97 |
| ALC pre-CAR T | 0.35 | 0.094 | 0.10 | 1.19 |
| Hematocrit pre-CAR T | 0.89 | **0.013** | 0.81 | 0.98 |
| Platelet count pre-CAR T | 0.08 | **0.002** | 0.02 | 0.40 |
| LDH pre-CAR T | 7.08 | **0.018** | 1.39 | 35.97 |
| Creatinine clearance pre-CAR T | 0.29 | 0.054 | 0.08 | 1.02 |
| Bulky disease | 0.32 | **0.016** | 0.13 | 0.81 |
| Product of diameters of the bigger lesion | 2.13 | **0.025** | 1.10 | 4.14 |
| Bigger lesion in extranodal site | 0.99 | 0.981 | 0.33 | 2.96 |
| Responsiveness to immediate pre-CAR therapy | 1.27 | 0.641 | 0.46 | 3.54 |
| ECOG Performance status | 0.52 | 0.246 | 0.17 | 1.57 |

***Post treatment***

| CRS | 1.56 | 0.480 | 0.46 | 5.32 |
| --- | --- | --- | --- | --- |
| CRS grade 3-4 | 4.77 | **0.007** | 1.53 | 14.89 |
| ICANS | 0.84 | 0.694 | 0.35 | 2.03 |
| ICANS grade 3-4 | 1.66 | 0.331 | 0.60 | 4.57 |
| Response at day +28 | 1.56 | 0.480 | 0.46 | 5.32 |
| Therapy after CAR T | 4.77 | **0.007** | 1.53 | 14.89 |

**Supplemental Table 6. Univariate analysis for factors impacting PFS**

***Pre-treatment***

| **Variable** | **HR** | **P value** | **HR (95% CI)** | |
| --- | --- | --- | --- | --- |
| Age | 1.00 | 0.895 | 0.97 | 1.03 |
| Sex | 1.48 | 0.367 | 0.63 | 3.50 |
| DH/TH | 0.84 | 0.671 | 0.38 | 1.86 |
| Diagnosis | 1.34 | 0.512 | 0.56 | 3.21 |
| Previous lines of treatment | 1.02 | 0.837 | 0.85 | 1.23 |
| Previous ASCT | 1.16 | 0.735 | 0.49 | 2.75 |
| Months to ASCT to CAR T | 0.99 | 0.444 | 0.96 | 1.02 |
| First line: R-CHOP vs R-EPOCH | 0.66 | 0.454 | 0.22 | 1.97 |
| First line: R-CHOP vs other | 1.02 | 0.983 | 0.14 | 7.72 |
| Months from first line to CAR T | 1.00 | 0.493 | 0.99 | 1.01 |
| Primary refractory disease | 1.18 | 0.687 | 0.52 | 2.69 |
| Time to relapse after first line | 1.04 | **0.014** | 1.01 | 1.08 |
| Weeks from last therapy to LA | 0.99 | 0.394 | 0.97 | 1.01 |
| Weeks from last therapy to LD | 0.97 | 0.898 | 0.58 | 1.62 |
| Bridging therapy | 0.83 | 0.617 | 0.39 | 1.75 |
| Novel drugs before CAR T | 0.86 | 0.723 | 0.38 | 1.94 |
| Previous use of polatuzumab vedotin | 2.31 | 0.213 | 0.62 | 8.63 |
| Previous infusion of a CAR T product | 0.37 | 0.109 | 0.11 | 1.25 |
| Number of bridging cycles | 1.01 | 0.972 | 0.53 | 1.93 |
| WBC pre-CAR T | 0.73 | 0.581 | 0.25 | 2.19 |
| ANC pre-CAR T | 0.95 | 0.922 | 0.37 | 2.45 |
| ALC pre-CAR T | 0.52 | 0.195 | 0.19 | 1.40 |
| Hematocrit pre-CAR T | 0.92 | **0.032** | 0.86 | 0.99 |
| Platelet count pre-CAR T | 0.29 | 0.064 | 0.08 | 1.08 |
| LDH pre-CAR T | 4.18 | **0.038** | 1.08 | 16.16 |
| Creatinine clearance pre-CAR T | 0.57 | 0.361 | 0.17 | 1.91 |
| Bulky disease | 1.29 | 0.641 | 0.44 | 3.74 |
| Product of diameters of the bigger lesion | 0.39 | **0.014** | 0.18 | 0.83 |
| Bigger lesion in extranodal site | 1.07 | 0.873 | 0.46 | 2.53 |
| Responsiveness to immediate pre-CAR therapy | 1.54 | 0.359 | 0.61 | 3.85 |
| ECOG Performance status | 0.72 | 0.496 | 0.28 | 1.84 |

***Post treatment***

| CRS | 1.04 | 0.945 | 0.39 | 2.75 |
| --- | --- | --- | --- | --- |
| CRS grade 3-4 | 2.38 | 0.115 | 0.81 | 6.98 |
| ICANS | 0.73 | 0.413 | 0.34 | 1.55 |
| ICANS grade 3-4 | 0.91 | 0.851 | 0.35 | 2.40 |
| Response at day +28 | 1.04 | 0.945 | 0.39 | 2.75 |
| Therapy after CAR T | 2.38 | 0.115 | 0.81 | 6.98 |

**Supplemental Table 7. Univariate analysis for factors impacting post-CAR T response at day+28**

***Pre-treatment***

| **Variable** | **HR** | **P value** | **HR (95% CI)** | |
| --- | --- | --- | --- | --- |
| Age | 1.02 | 0.361 | 0.98 | 1.07 |
| Sex | 1.12 | 0.844 | 0.35 | 3.52 |
| DH/TH | 2.73 | 0.101 | 0.82 | 9.27 |
| Diagnosis | 0.84 | 0.783 | 0.24 | 3.17 |
| Previous lines of treatment | 0.86 | 0.375 | 0.62 | 1.20 |
| Previous ASCT | 0.65 | 0.525 | 0.16 | 2.30 |
| Months to ASCT to CAR T | 1.00 | 0.915 | 0.96 | 1.04 |
| First line: R-CHOP vs R-EPOCH | 1.75 | 0.516 | 0.37 | 12.72 |
| First line: R-CHOP vs other | 0.25 | 0.273 | 0.01 | 2.81 |
| Months from first line to CAR T | 1.01 | 0.272 | 1.00 | 1.02 |
| Primary refractory disease | 1.29 | 0.683 | 0.39 | 4.74 |
| Time to relapse after first line | 1.00 | 0.664 | 0.97 | 1.02 |
| Weeks from last therapy to LA | 1.04 | 0.106 | 1.00 | 1.10 |
| Weeks from last therapy to LD | 1.27 | 0.537 | 0.57 | 2.90 |
| Bridging therapy | 1.11 | 0.850 | 0.37 | 3.47 |
| Novel drugs before CAR T | 1.00 | 1.000 | 0.31 | 3.11 |
| Previous use of polatuzumab vedotin | 0.30 | 0.213 | 0.04 | 1.94 |
| Previous infusion of a CAR T product | 4.35 | 0.243 | 0.39 | 97.53 |
| Number of bridging cycles | 1.00 | 1.000 | 0.35 | 2.87 |
| WBC pre-CAR T | 1.56 | 0.595 | 0.29 | 8.43 |
| ANC pre-CAR T | 1.23 | 0.767 | 0.30 | 4.80 |
| ALC pre-CAR T | 6.01 | 0.051 | 1.09 | 42.76 |
| Hematocrit pre-CAR T | 1.17 | **0.021** | 1.03 | 1.35 |
| Platelet count pre-CAR T | 10.33 | **0.043** | 1.19 | 119.21 |
| LDH pre-CAR T | 0.08 | **0.047** | 0.00 | 0.78 |
| Creatinine clearance pre-CAR T | 0.65 | 0.716 | 0.03 | 5.48 |
| Bulky disease | 0.44 | 0.289 | 0.09 | 2.09 |
| Product of diameters of the bigger lesion | 5.16 | **0.007** | 1.60 | 17.95 |
| Bigger lesion in extranodal site | 0.87 | 0.828 | 0.25 | 3.27 |
| Responsiveness to immediate pre-CAR therapy | 1.66 | 0.494 | 0.42 | 8.26 |

***Post treatment***

| CRS | 1.15 | 0.828 | 0.31 | 4.01 |
| --- | --- | --- | --- | --- |
| CRS grade 3-4 | 0.30 | 0.206 | 0.04 | 1.95 |
| ICANS | 2.12 | 0.193 | 0.70 | 6.83 |
| ICANS grade 3-4 | 0.81 | 0.788 | 0.18 | 4.33 |
| Therapy after CAR T | 2.19 | 0.369 | 0.37 | 12.99 |

Supplemental tables 3-5. DH/TH: double hit/triple hit mutated. ASCT: autologous stem cell transplant. LA: leukoapheresis. LD: lymphodepletion. WBC: white blood cell count. ANC: absolute neutrophils count. ALC: absolute lymphocyte count. LDH: lactate dehydrogenase. CRS: cytokine release syndrome. ICANS: Immune effector cell-associated neurotoxicity syndrome. Responsiveness to immediate pre-CAR therapy and response at day +28: complete response and partial response (responders) vs. stable disease and progression disease (non-responders).

**Supplemental table 8. Association of pre-CAR T treatment with post-CAR T OS. PFS. and response to CAR T in a multivariable analysis containing “product of diameters”**

| **Prognostic factors** | **OS** | | **PFS** | | **ORR** | |
| --- | --- | --- | --- | --- | --- | --- |
|  | HR (95% CI) | P | HR (95% CI) | P | HR (95% CI) | P |
| Product of diameters | 1.48 (0.65-3.38) | .349 | 1.55 (0.87-2.74) | .134 | 0.20 (0.05-0.06) | **.016** |
| Low HCT pre-CAR T | 0.82 (0.71-0.94) | **.004** | 0.93 (0.85-1.01) | .086 | 1.21 (1.02-1.49) | **.045** |
| Elevated LDH pre-CAR T | 6.86 (1.34-35.20) | **.021** | 2.94 (0.68-12.72) | .150 | 0.28 (0.01-5.35) | .433 |
| Bridging therapy | 0.32 (0.10-1.00) | **.050** | 0.66 (0.27-1.60) | .353 | 3.39 (0.71-13.38) | .142 |
| DH/TH | 8.26 (2.03-33.62) | **.003** | 1.34 (0.55-3.28) | .525 | 1.80 (0.44-7.36) | .406 |

Supplemental table 6: P values in bold are statistically significant. OS: overall survival. PFS: progression free survival. ORR: overall response rate. HR. hazard ratio. HCT: hematocrit. LDH. lactate dehydrogenase. DH/TH/only c-myc: double hit/triple hit mutated.
